# Pulmonary Coccidioidomycosis Occurring in a Patient Treated With Acalabrutinib for Chronic Lymphocytic Leukemia

**DOI:** 10.7759/cureus.83026

**Published:** 2025-04-26

**Authors:** Zahra Gafarzadeh, Cyril Gaultier, Shiva Salmasi, Ruba Alchaikh Hassan, Constantin A Dasanu

**Affiliations:** 1 Internal Medicine, Eisenhower Medical Center, Rancho Mirage, USA; 2 Infectious Disease, Eisenhower Medical Center, Rancho Mirage, USA; 3 Oncology and Hematology, Lucy Curci Cancer Center, Eisenhower Medical Center, Rancho Mirage, USA

**Keywords:** acalabrutinib, bruton tyrosine kinase inhibitor, chronic lymphocytic leukemia, oncology, pulmonary coccidioidomycosis

## Abstract

Acalabrutinib is a Bruton tyrosine kinase inhibitor (BTKi) approved for use in the treatment of chronic lymphocytic leukemia (CLL). Herein, we present a patient successfully treated with reduced-dose acalabrutinib for CLL, with pre-existing hypogammaglobulinemia-type immunoglobulin G (IgG) and immunoglobulin M (IgM). Twenty-four months into therapy, he developed a right upper lobe infiltrate due to pulmonary coccidioidomycosis; the Naranjo causality assessment score was 4 (probable). The patient received monthly intravenous IG (IVIG) infusions and antifungal therapy, with significant clinical improvement. Acalabrutinib was restarted, along with close clinical monitoring. The extent to which invasive fungal infections can be attributed to acalabrutinib alone is not always straightforward due to the presence of immune defects associated with CLL, endemic zip codes, and a prior exposure to ibrutinib. Physicians should remain vigilant in assessing and managing invasive fungal infections in these patients in order to optimize patient safety and clinical outcomes.

## Introduction

Bruton tyrosine kinase inhibitors (BTKis) are a key class of targeted therapies for B-cell malignancies such as chronic lymphocytic leukemia (CLL) and mantle cell lymphoma (MCL). Acalabrutinib, a second-generation BTKi, improves progression-free and overall survival in CLL and offers a more favorable side-effect profile than first-generation agents [1,2].

Although BTKis increased infection risk, invasive fungal infections are relatively uncommon. Most reported cases involve ibrutinib, with Aspergillus species being the predominant pathogen [3-6]. Fungal infections associated with acalabrutinib are rarely described, and cases involving Coccidioides species are exceedingly rare. Coccidioides, endemic to the southwestern US and parts of Latin America, typically causes mild respiratory illness in immunocompetent hosts but can lead to severe disease in immunocompromised patients.

We report a rare case of pulmonary coccidioidomycosis in a CLL patient treated with acalabrutinib, emphasizing the need to consider regional fungal infections in patients receiving BTKis. This case highlights the importance of geographic context, diagnostic challenges, and infection risk management in immunosuppressed individuals.

## Case presentation

A 72-year-old Caucasian man, with a known history of CLL, presented to the emergency room (ER) in August 2023 with a one-week history of progressively worsening flu-like symptoms, generalized weakness, shortness of breath, loss of appetite, unintentional weight loss, and maculopapular skin lesions on both palms.

**Figure 1:**
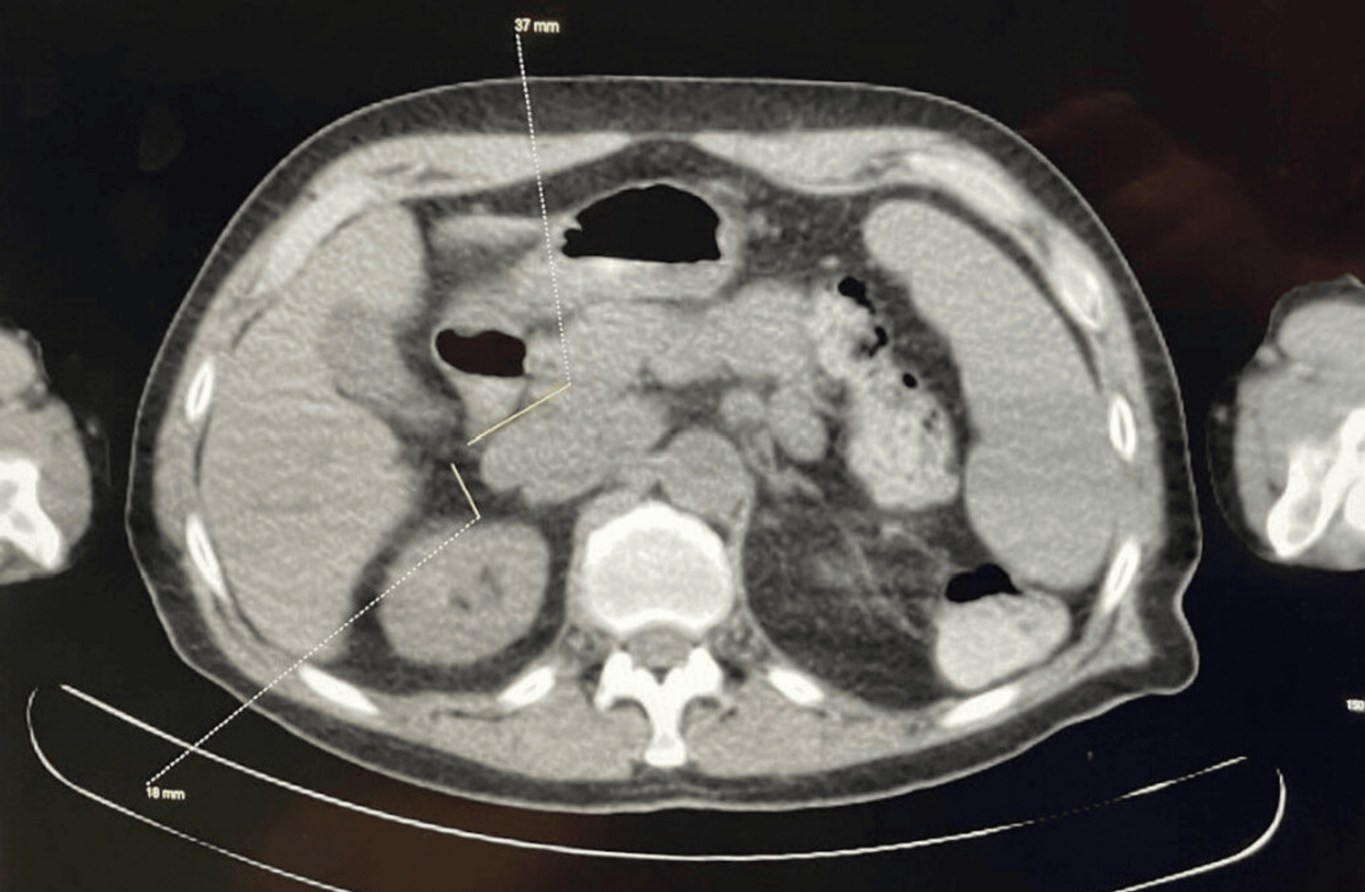
PET-CT scan showing low-grade metabolic activity in an enlarged anterior portacaval node.

He was initially diagnosed with CLL in 2021 (Rai stage IA) after presenting with cervical lymphadenopathy and leukocytosis (WBC count: 29,000 cells/mm³; reference range: 3,800-10,800 cells/mm³). Diagnosis was confirmed via a bone marrow biopsy and fluorescence in situ hybridization (FISH) studies. PET-CT at the time revealed low-grade metabolic activity in multiple lymph nodes above and below the diaphragm, without evidence of Richter transformation (Figure 1).

He was started on ibrutinib (March-July 2021), which was discontinued due to severe arthralgias. He was then initiated on acalabrutinib 100 mg twice daily in August 2021 but again experienced significant arthralgias and myalgias. The dose was subsequently reduced to 100 mg daily, with stable disease control. His medical history was notable for hypogammaglobulinemia (low immunoglobulin G (IgG) and immunoglobulin M (IgM)), multiple non-melanoma skin cancers, hyperlipidemia, mild chronic kidney disease, and insomnia. He was an ex-smoker with a 50-pack-year history, consumed alcohol occasionally, and used marijuana daily for sleep. He denied recent travel or known exposure to infectious sources.

**Figure 2:**
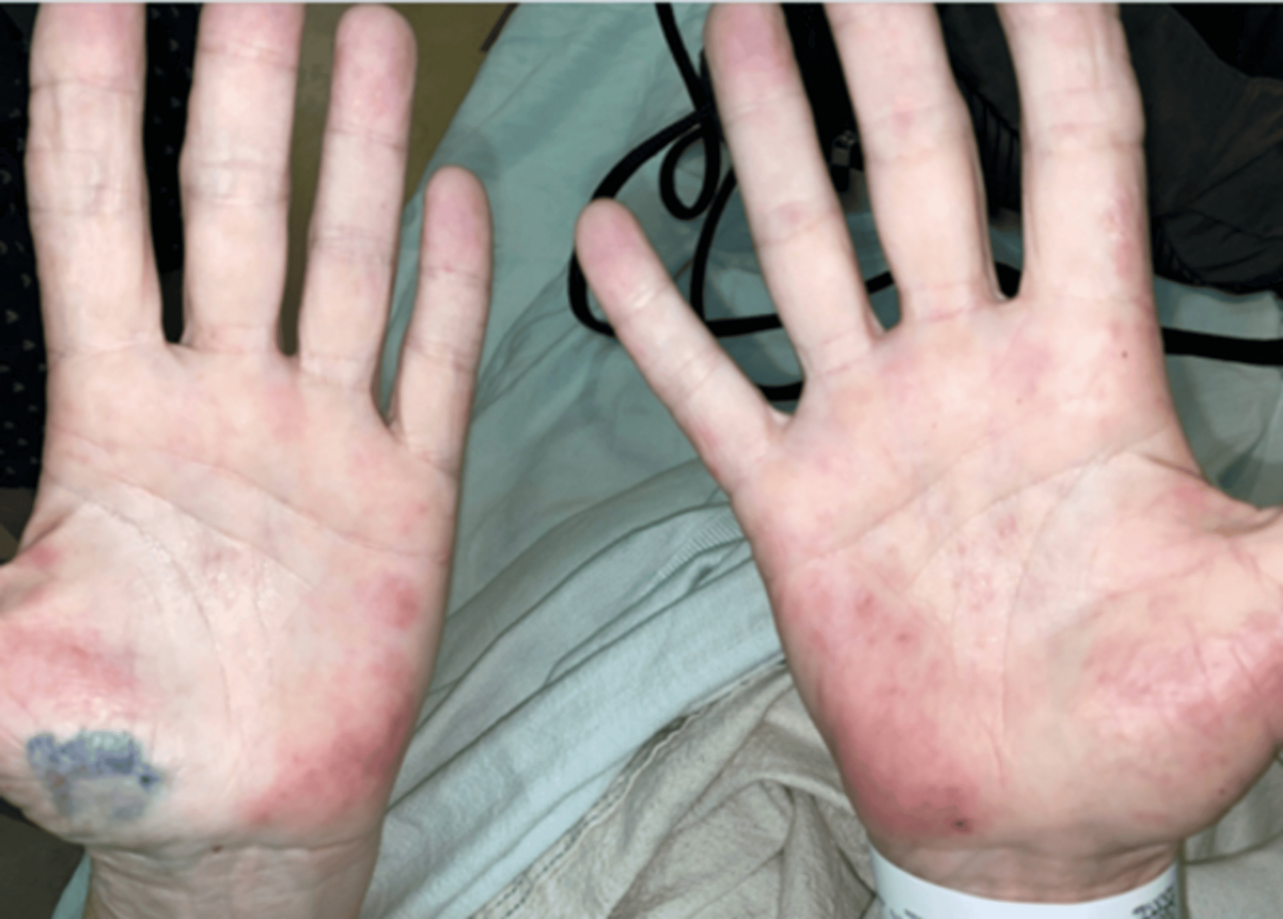
Erythematous, maculopapular lesions on the palms bilaterally in the index patient. The skin is affected in up to 50% of cases during the acute phase of coccidioidomycosis, most commonly in the form of a non-pruritic papular rash, erythema nodosum, and erythema multiforme and can present as palmar skin lesions as well.

Upon initial ER evaluation, he was tachypneic and tachycardic with coarse rales in the right upper lung field. Chest X-ray showed right upper lobe consolidation, and he was admitted with a presumptive diagnosis of community-acquired pneumonia. He received intravenous cefepime and azithromycin and was started on monthly intravenous IG (IVIG) therapy for hypogammaglobulinemia. Acalabrutinib was continued. Three weeks later, he was readmitted with worsening fatigue, low-grade fever, nausea, anorexia, weight loss, and persistent shortness of breath. He remained hemodynamically stable and oxygenating well on room air. Physical examination revealed a non-blanching maculopapular rash on both palms (Figure 2).

**Figure 3:**
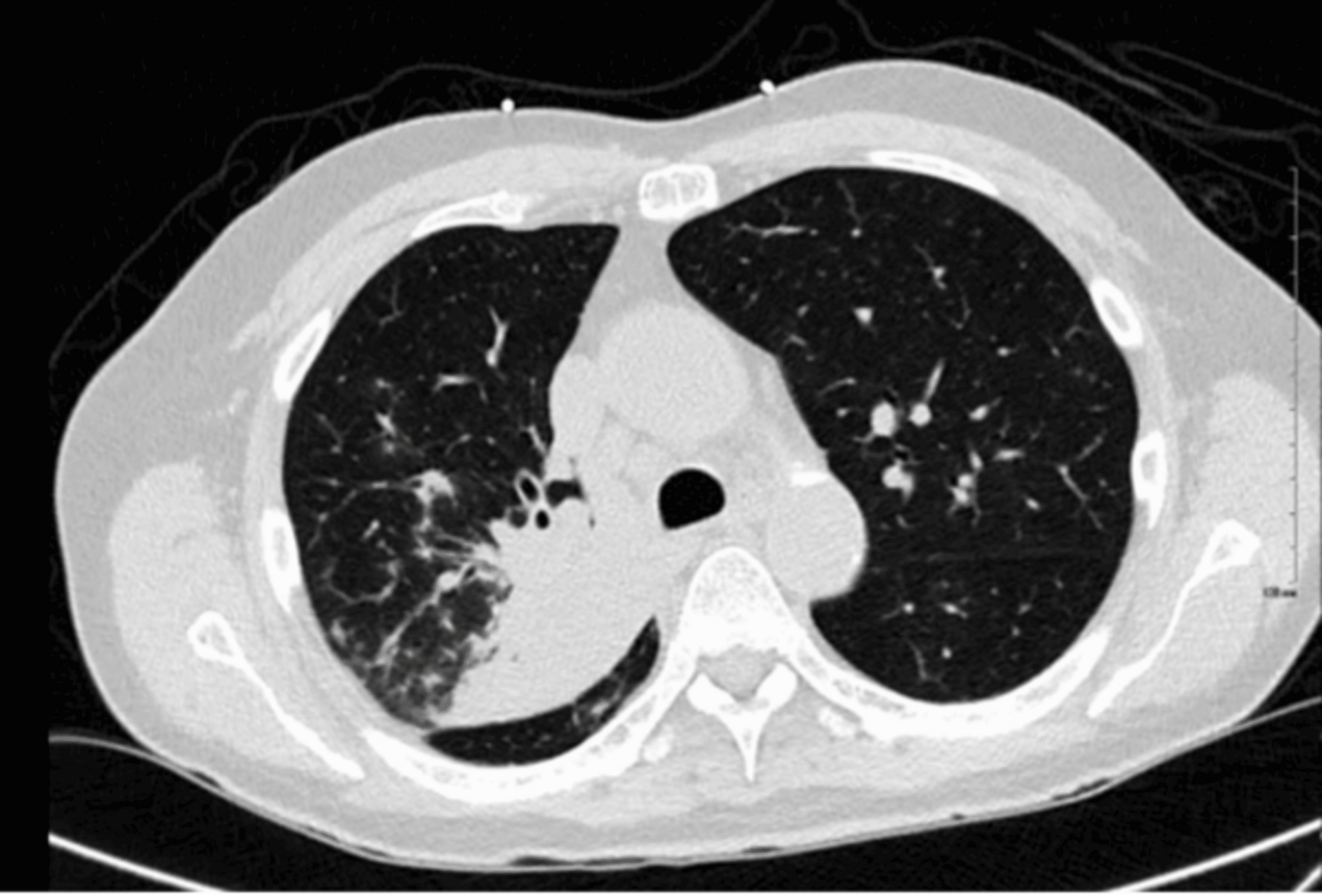
CT scan of the chest without contrast demonstrated a confluent right upper lobe infiltrate with posterior segment consolidation.

Repeat chest X-ray showed new, ill-defined right perihilar opacities. Labs were notable for elevated C-reactive protein (CRP) (5.8 mg/dL; ref <1.0) and erythrocyte sedimentation rate (ESR) (77 mm/hr; ref 0-20). The respiratory panel was negative. Non-contrast chest CT revealed confluent right upper lobe infiltrates with posterior segment consolidation (Figure 3).

Empiric antibiotics were broadened to cefepime and vancomycin. Due to the palmar rash and systemic symptoms, the differential diagnosis included syphilis, Rocky Mountain spotted fever, and infective endocarditis. Doxycycline was initiated. Blood cultures were negative, rapid Methicillin-resistant Staphylococcus aureus (MRSA) screen was negative, and vancomycin was discontinued. TTE showed no valvular vegetations. Syphilis serology was negative. Acalabrutinib was held during hospitalization after discussion with oncology. By hospital day three, the patient’s condition improved clinically, and he was discharged on oral levofloxacin and doxycycline, with outpatient follow-up arranged.

Subsequently, Coccidioides serology via immunodiffusion with complement fixation returned positive for both IgM and IgG antibodies (titer 1:4). Serum 1,3 beta-D-glucan was 52 pg/mL (ref <60 pg/mL). Additional testing for other pathogens, including Streptococcus pneumoniae, Legionella, Mycoplasma pneumoniae, Epstein-Barr virus (EBV), cytomegalovirus (CMV), Aspergillus, Blastomyces, Histoplasma, and Strongyloides, was negative. Given the patient’s clinical presentation, positive serologic findings, and exclusion of other etiologies, a presumptive diagnosis of pulmonary coccidioidomycosis was made.

Outcome and follow-up

At initial presentation, the patient was started on empiric antibiotic therapy for presumed community-acquired pneumonia, as coccidioidomycosis was not initially suspected. Given the time required for coccidioidal serology results, antifungal therapy was not initiated immediately. Once the serology returned positive for Coccidioides antibodies, and the patient had already begun to show clinical and radiographic improvement, antifungal treatment was started at a prophylactic dose. This approach aimed to address any ongoing low-grade fungal infection and to minimize the risk of opportunistic fungal infections in the context of continued BTKi therapy.

**Figure 4:**
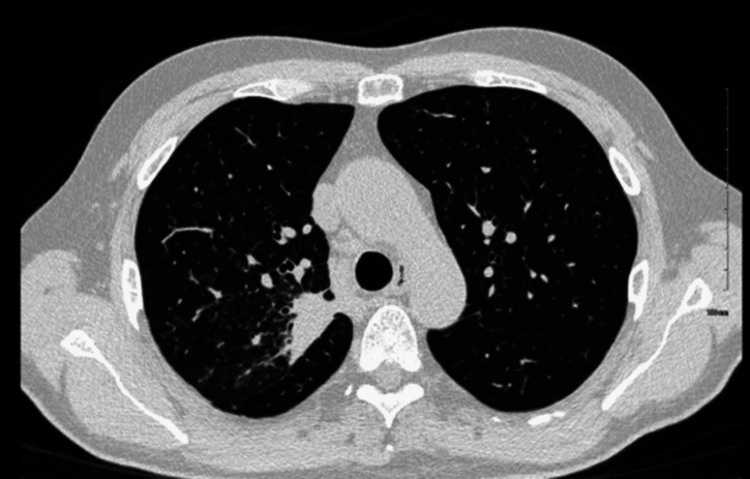
Interval decrease in the size of the previously seen consolidation in the posterior aspect of the right upper lobe (RUL) compatible with a resolving infectious/inflammatory process.

At the time of diagnosis, the patient’s clinical condition had returned to baseline. Serum 1,3 beta-D-glucan decreased to <31 pg/mL, complement fixation (CF) titers normalized, and CRP returned to normal levels. Follow-up chest CT in January 2024 demonstrated significant regression of the pulmonary infiltrates that were initially seen in August-September 2023 (Figure 4).

To reduce infectious risk, secondary prophylaxis with fluconazole 100 mg orally daily was initiated, and monthly IVIG infusions were continued starting September 2023. Acalabrutinib was cautiously reintroduced at a reduced dose of 100 mg orally daily, balancing the risk of CLL progression with the potential for infection. Currently, the patient remains clinically asymptomatic and is under close surveillance by both infectious disease and hematology-oncology teams, with regular monitoring of laboratory parameters and imaging studies.

## Discussion

Acalabrutinib is a highly selective second-generation covalent BTKi approved for adults with previously untreated or relapsed/refractory CLL [1,2]. BTK plays a pivotal role in B-cell receptor signaling. Activation of the B-cell receptor triggers the phosphorylation of BTK, thereby initiating pathways crucial for B-cell proliferation and survival [7]. Inactivating the human BTK gene results in X-linked agammaglobulinemia, an inherited immune disorder characterized by the absence of mature B lymphocytes and IG production [7]. BTK received attention as a potential therapeutic target due to its expression in numerous B-cell malignancies, along with its anti-apoptotic function [8]. BTKis bind to BTK, thereby inducing apoptosis in B-cell malignancies.

Clinical trials have shown that acalabrutinib is better tolerated due to being more selective compared to ibrutinib (a first-generation BTKi), resulting in minimal off-target activity across other kinases [9]. Although BTK inhibitors are associated with a heightened risk of infections of any grade, fungal infections are seldom reported in clinical trials. Ibrutinib, as the first agent in this class, remains the most extensively studied and provides ample data in terms of its side effect profile. Consequently, the majority of reported fungal infections are linked with ibrutinib use in observational studies and case reports, with Aspergillus spp. being the predominant causative agent [3-6].

Pathophysiology of fungal infections seen with BTKis involves a complex immunodeficiency affecting a broad range of immune cells in the adaptive and innate immune system. Several potential mechanisms underlying increased susceptibility include impairments in macrophage and neutrophil antifungal functions, due to off-target activity on these cells [10,11]. Acalabrutinib exhibits enhanced binding selectivity with less activity on non-target cell types. Despite not inhibiting interleukin-2-inducible kinase critical for CD4 T-cell signaling, cases of disseminated fungal infections have also been documented with this agent [12]. This suggests that susceptibility to these infections is not solely attributable to the off-target effects of BTK inhibitors on T-cells, macrophages, and neutrophils but also involves B-cell pathways.

Given the patient's compromised immune system due to CLL and acalabrutinib treatment, a more rapid disease progression would have been anticipated in our patient. The number of reported fungal infections with acalabrutinib is less than that seen with ibrutinib. However, in the ELEVATE-RR trial, a phase 3 randomized trial comparing ibrutinib to acalabrutinib for the treatment of CLL, there were more fungal infections reported in the acalabrutinib group. Fungal opportunistic infections occurred in 3.8% of patients treated with acalabrutinib (PJP (n = 5); broncho-pulmonary aspergillosis (n = 3); and Aspergillus infection, cerebral aspergillosis, and cryptococcosis (one patient each)) and 1.9% of patients treated with ibrutinib (esophageal candidiasis (n = 2); and coccidioidomycosis, cerebral aspergillosis, and broncho-pulmonary aspergillosis (one patient each)) [9]. In a pooled analysis of safety data from clinical trials conducted by Furman et al. [13] and including 1,040 patients with B-cell malignancies treated with acalabrutinib, 63 fungal infections were identified, including three fatal infections. Among cases classified as serious, four cases were due to Aspergillus, one was due to Candida, and one case was due to Cryptococcus. A large safety analysis of 610 patients by Furman et al. [13] on acalabrutinib monotherapy described only four fungal infections, and one case report of disseminated cryptococcal infection was identified in the literature [14]. Another case was reported in a patient treated with acalabrutinib plus obinutuzumab who developed cerebral aspergillosis [15]. To our knowledge, this is the first report of coccidioidomycosis in a patient treated with single-agent acalabrutinib.

Coccidioidomycosis, also referred to as Valley fever, is an invasive dimorphic fungal infection caused by a soil-borne fungus. The fungi are found in the arid desert soils of the southwestern US, as well as in parts of Mexico and Central and South America. This infectious disease presents a spectrum from asymptomatic to pulmonary or disseminated infection. While most infections resolve without medical intervention, immunosuppressed patients are prone to developing more severe diseases. The mechanism by which acalabrutinib increases susceptibility to coccidioidomycosis requires additional study. Although T-cells, particularly Th1, Th17, and interferon-gamma production, are crucial for controlling the infection and preventing its dissemination beyond the lungs [16], studies suggest that B-cells have a protective role in response to Coccidioides, with the IgG antibody being the predominant isotype observed in humoral-mediated responses [17]. Therefore, we postulate that the less severe disease observed in our patient could be attributed to IVIG therapy and the lack of inhibition of interleukin-2 inducible kinase by acalabrutinib due to its enhanced selectivity, which is critical for CD4+ T-cell signaling.

It is difficult to establish the extent to which the risk of opportunistic infections, particularly invasive fungal infections, in patients receiving BTKis, is attributable to the antineoplastic drug alone. This complexity arises from the presence of various factors that heighten infection risk in patients, along with potential immune defects associated with the underlying CLL. The risk of invasive fungal infections appears to be most pronounced in CLL patients who have undergone prior antineoplastic therapy or received concurrent corticosteroids alongside the BTKi [18]. In untreated SLL/CLL, the vast majority of infections are bacterial. By contrast, viral and fungal opportunistic infections are rare and occur predominantly in advanced stages of the disease or in the context of severe neutropenia [19]. In our patient, the Naranjo nomogram score of 4 indicates acalabrutinb as a possible culprit [20] (Table 1). Our patient’s CLL was in remission with acalabrutinib, and there was no neutropenia at presentation, which makes it conceivable that acalabrutinib might have been involved. However, it is important to acknowledge the potential contribution of his residing in an endemic area, the underlying hematologic disorder, and prior exposure to ibrutinib.

**Table 1:** The Naranjo adverse drug reaction (ADR) probability scale questionnaire in the index patient. The Naranjo criteria classify the probability that an adverse event is related to drug therapy based on a list of weighted questions, which examine factors such as the temporal association of drug administration and event occurrence, alternative causes for the event, drug levels, dose-response relationships, and previous patient experience with the medication. The ADR is assigned to a probability category from the total score as follows: definite if the overall score is 9 or greater, probable for a score of 5–8, possible for 1–4, and doubtful if the score is 0. The Naranjo criteria do not take into account drug-drug interactions. Drugs are evaluated individually for causality, and points are deducted if another factor may have resulted in the adverse event, thereby weakening the causal association. Source: Ref [20]

To assess the adverse drug reaction, please answer the following questionnaire and give the pertinent score.	Yes	No	Do not know	Score
1. Are there previous conclusive reports on this reaction?	+1	0	0	+1
2. Did the adverse event appear after the suspected drug was administered?	+2	-1	0	+2
3. Did the adverse event improve when the drug was discontinued, or a specific antagonist was administered?	+1	0	0	+1
4. Did the adverse event reappear when the drug was re administered?	+2	-1	0	0
5. Are there alternative causes that could on their own have caused the reaction?	-1	+2	0	-1
6. Did the reaction reappear when a placebo was given?	-1	+1	0	0
7. Was the drug detected in the blood or other fluids in concentrations known to be toxic?	+1	0	0	0
8. Was the reaction more severe when the dose was increased or less severe when the dose was decreased?	+1	0	0	0
9. Did the patient have a similar reaction to the same or similar drugs in any previous exposure?	+1	0	0	0
10. Was the adverse event confirmed by any objective evidence?	+1	0	0	+1
Score: 4				

## Conclusions

While BTKis have significantly advanced the treatment of hematologic malignancies, clinicians must remain vigilant about the risk of opportunistic fungal infections, particularly in real-world settings, where such complications may emerge outside the scope of clinical trials. This case underscores the importance of maintaining a high index of suspicion for endemic mycoses such as coccidioidomycosis in patients receiving BTKis, especially in geographic regions where these pathogens are prevalent. Prompt recognition, timely serologic testing, and early initiation of supportive therapy - including antifungal prophylaxis - are critical to optimizing patient outcomes. Although this is a single case, it highlights the need for proactive infection risk assessment and suggests that surveillance strategies may be beneficial in similar clinical scenarios. Further research is warranted to better understand infection patterns and guide evidence-based management in this patient population.
